# Density‐induced social stress alters oxytocin and vasopressin activities in the brain of a small rodent species

**DOI:** 10.1111/1749-4877.12467

**Published:** 2020-08-16

**Authors:** Shuli HUANG, Guoliang LI, Yongliang PAN, Mingjing SONG, Jidong ZHAO, Xinrong WAN, Charles J. KREBS, Zuoxin WANG, Wenxuan HAN, Zhibin ZHANG

**Affiliations:** ^1^ State Key Laboratory of Integrated Pest Management Institute of Zoology Chinese Academy of Sciences Beijing China; ^2^ CAS Center for Excellence in Biotic Interactions University of Chinese Academy of Sciences Beijing China; ^3^ School of Medicine Huzhou University Huzhou China; ^4^ Key Laboratory of Human Disease Comparative Medicine National Health Commission of China (NHC) Beijing Engineering Research Center for Experimental Animal Models of Human Diseases Institute of Laboratory Animal Science Peking Union Medicine College Chinese Academy of Medical Science Beijing China; ^5^ Department of Zoology University of British Columbia Vancouver British Columbia Canada; ^6^ Department of Psychology and Program in Neuroscience Florida State University Tallahassee Florida USA; ^7^ College of Resources and Environmental Sciences China Agricultural University Beijing China

**Keywords:** aggression behavior, density‐dependent stress, oxytocin (OT), social stress, vasopressin (AVP)

## Abstract

It is known that social stress could alter oxytocin (OT) and arginine‐vasopressin (AVP) expression in specific regions of brains which regulate the aggressive behavior of small rodents, but the effects of density‐induced social stress are still unknown. Brandt's voles (*Lasiopodomys brandtii*) are small herbivores in the grassland of China, but the underlying neurological mechanism of population regulation is still unknown. We tested the effects of housing density of Brandt's voles on OT/AVP system with physical contact (allowing aggression) and without physical contact (not allowing aggression) under laboratory conditions. Then, we tested the effects of paired‐aggression (no density effect) of Brandt's voles on OT/AVP system under laboratory conditions. We hypothesized that high density would increase aggression among animals which would then increase AVP but reduce OT in brains of animals. Our results showed that high housing density induced more aggressive behavior. We found high‐density‐induced social stress (with or without physical contact) and direct aggression significantly increased expression of mRNA and protein of AVP and its receptor, but decreased expression of mRNA and protein of OT and its receptor in specific brain regions of voles. The results suggest that density‐dependent change of OT/AVP systems may play a significant role in the population regulation of small rodents by altering density‐dependent aggressive behavior.

## Graphical abstract

High housing density induced social stress (with or without physical contact) and direct aggression significantly increased expression of mRNA and protein of arginine‐vasopressin (AVP) and its receptor, but decreased expression of mRNA and protein of oxytocin (OT) and its receptor in specific brain regions of Brandt’s voles (*Lasiopodomys brandtii*), suggesting that density‐dependent change of OT/AVP systems may play a significant role in the population regulation of small rodents by altering density‐dependent aggressive behavior.

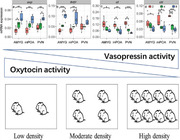

## INTRODUCTION

Oxytocin (OT) and arginine‐vasopressin (AVP) are two well‐known neuropeptides that have been implicated in a variety of cognitive and behavioral functions, including social recognition, aggression, parental care, mating, learning, and memory (Trainor *et al*. 2003; Goodson & Evans 2004; De Vries & Panzica 2006). OT and AVP are mainly synthesized in the paraventricular nucleus (PVN) and supraoptic nucleus of the hypothalamus (SON), and act in several brain areas such as the anterior hypothalamus (AH), medial preoptic area (MPOA), bed nucleus of the stria terminalis, and medial amygdala (MeA) in the brain (Albers *et al*. 1988; Ferris *et al*. 1997; Caldwell & Albers 2003, 2004). Previous studies demonstrated that OT, AVP, and their receptors (i.e. OTR and V1aR) are involved in the regulation of aggressive behavior in mammals (see below). For example, a high level AVP is associated with intensified aggression and flank marking behaviors in male golden hamsters (*Mesocricetus auratus*) (Ferris *et al*. 1984; Ferris & Potegal 1988). OT injections in the MeA and BST in the brain significantly reduced the aggression frequency of female rats (Rattus norvegicus) (Consiglio *et al*. 2005). Injections of OTR antagonists and V1aR agonists increased the attack frequency of pregnant female rats to intruders (Lubin *et al*. 2003). Injection of AVP in the MPOA‐AH region of Mauratus increased its aggressive behavior, but injection of V1aR antagonists decreased its aggressive behavior (Hennessey *et al*. 1994). Because high‐density population of small rodents is often characterized by increaseing of aggression among individuals, we speculated that density‐dependent aggressive behavior is regulated by the OT/AVP systems in small rodents.

There have been many studies on the relationships between stress and the neuroendocrine systems in animals, mostly focusing on the hypothalamic‐pituitary‐adrenal (HPA) axis system. Stress stimulates the HPA axis via PVN, which then results in the release of corticotropin‐releasing hormone (CRH) and AVP to stimulate the release of adrenocorticotropic hormone (ACTH) in the anterior pituitary gland. Plasma CORT, the stress hormone released from the adrenal cortex, acts on a feedback loop to negatively regulate the release of CRH and ACTH. ACTH also regulates the release of CRH, and then OT and AVP (Johnson *et al*. 1992). It has also been well documented that stress induces AVP syntheses in the PVN as well as their releases in selected brain areas (Johnson *et al*. 1992). Released AVP and OT have opposing effects: AVP facilitates stress responses whereas OT has buffering effects in reducing stress responses (Smith & Wang 2014). These data suggest that stress‐associated releases of OT/AVP could mediate the aggressive behavior of animals.

Although the close relationship between social stress and OT/AVP systems has been shown, the effects of density‐dependent social stress on OT/AVP system remains unexplored. It is recognized that high density often increases aggression behaviors of animals (Christian 1950; Wynne‐Edwards 1962; Chitty 1967). Thus, we hypothesized that high density‐induced stress (e.g. direct aggression, crowding stress) could increase AVP but reduce OT through physical contact (e.g. through aggression stress) or through non‐physical contact (e.g. though odor, vision and sound stress). Based on our hypothesis, we have the following predictions: (1) High‐density stress should increase the frequency of aggressive behavior; (2) high‐density stress with or without physical contact should increase the expression level of AVP and its receptor, but decrease the expression level of OT and its receptor; (3) aggression increase the expression level of AVP and its receptor, but decrease the expression level of OT and its receptor.

Brandt's voles (*Lasiopodomys brandtii*) are small social herbivores and are widely distributed in the grassland of Inner Mongolia, China, and Mongolia. Their populations oscillate greatly across different years (Zhang *et al*. 2003). Our previous studies have demonstrated that fluctuations in population densities of Brandt's vole are caused by the combined effects of the extrinsic and intrinsic components (Li *et al*. 2016). The purpose of the present study is to test the above three predictions based on our hypothesis that high density could increase AVP but reduce OT through both physical and non‐physical contact under laboratory conditions.

## MATERIALS AND METHODS

### The study subject and sites

The Brandt's voles were used in this study. The experiment 1 and 3 were conducted in the field station which is located in the Maodeng Pasture, Xilinhaote city, Inner Mongolia, China (for details, see Li*et al*. 2016). Overwintering adult voles were captured in the field nearby the station and raised for 2 weeks under laboratory conditions in the station before the experiment. For keeping clean living conditions, the bedding was changed every week. The temperature of the house was about 24 °C with natural light cycle, ensuring the voles had a good state to acclimate to the environment of the house in 2 weeks. Experiment 2 was conducted in the Institute of Zoology, Chinese Academy of Sciences. The animals come from a colony of Brandt's voles in the Institute of Zoology. (The living condition was similar to that in field, where also the bedding was changed every week and the temperature of the house was 24 °C, but the light cycle was stable with day for 14 h and night 10 h.)

### Experiment 1: density experiment with physical contact

To test the density‐dependent associations between frequency of aggressive behavior and OT/AVP systems, density manipulation experiment with physical contact was conducted in the laboratory from early July to late August in 2016. Newborn mature voles with body weight were about 35–40 g captured from the grassland nearby our field station in Xilinhaote, Inner Mongolia. Voles in male–female pairs were housed in polypropylene cages (25.5 cm × 15 cm × 13.5 cm) under natural photoperiodic conditions and were allowed a 2‐weeks acclimation period before the experiments. After the acclimation period, voles were weighed and randomly assigned to low (1 male and 1 female), moderate (2 males and 2 females), and high density (4 males and 4 females) treatment groups (Fig. S1, Supporting Information). Each treatment group had 5 replicates. We determined this based on minimizing the animals used and the number of individuals (4–6 individuals/per box) raised under laboratory conditions. Voles of each treatment group were housed in a polypropylene cage (25.5 cm × 15 cm × 13.5 cm) under natural photoperiodic conditions for 4 weeks to simulate the chronic social stress of population density. Voles of each treatment group were marked at different body positions with hair dye so as to distinguish them from each other. At the end of experiment, the whole fresh brain samples were then stored in liquid nitrogen and delivered to our lab in Beijing.

To examine the relationship between housing densities and frequency of aggressive behaviors (aggressive frequency and duration) of Brandt's voles, we observed the behaviors of voles in different density treatment groups by the use of video cameras. Behavioral observation was conducted in 3 different time periods: late morning (0730−0900), around noon (1100−1230), late afternoon (1700–1830), and lasted for 3 successive days (at day 25, day 26, and day 27). Aggression is defined as hostile, injurious, or destructive behavior (Siever 2008) and the animals express aggression in qualitatively similar ways using biting as their main strategy to harm an opponent. The level of aggression can be measured by attack frequency and duration (Hashikawa *et al*. 2018).

### Experiment 2: density experiment without physical contact

To examine the density‐dependent effects on the OT/AVP system by excluding the effects of aggressive behavior, a laboratory density manipulative experiment without physical contact was conducted in 2018. The density intensity was measured by the number of social neighbors without physical contact. Each individual was separately housed in a wire mesh cage (25.5 cm × 15 cm × 13.5 cm). Cages were put together side by side to simulate the crowding condition. For the low‐crowding, medium‐crowding, and high‐crowding groups, 2 (1 × 1), 4 (2 × 2), and 25 (5 × 5) cages were put together, with an interval of 3 cm apart between neighboring cages (Fig. S2, Supporting Information; each individual of low‐crowding, medium‐crowding groups have 1 and 3 neighbors, respectively). For high‐crowding groups, we selected individuals with 8 neighbors. The low‐crowding and medium‐crowding groups had 4 and 3 replicates, respectively. The high‐crowding group had no replicates to minimize the number of animals used. In this experiment, voles were in visual, olfactory, auditory, and tactile contact with each other but were prevented from full body contact to exclude physical aggressive behavior. The experiment was carried out for 4 successive weeks. At the end of the experiment, the whole fresh brains of voles were collected and stored in liquid nitrogen.

### Experiment 3: aggression experiment

Density manipulation experiment contains two effects: aggressive behavior due to unfamiliar encounters and crowding due to shortage of space or stress of unfamiliar odor, noise, or vision. To examine the density‐dependent effects of aggressive behaviors on the OT/AVP system by excluding the crowding effects, a fighting experiment was conducted in the lab in 2017. Adult male voles were captured from the field. They were housed separately in cages and were provided with clean bedding as well as food and water. After 2 weeks of acclimation, two males of similar weight (55−60 g) were introduced into a new arena (50 cm × 45 cm × 40 cm) to perform fighting experiments. The behaviors of these two voles were recorded by camera for 5 min. All agonistic encounters took place between 0800 and 1000 for successive 21 days. There were 9 replicates in this fighting experiment. Another 9 males were taken as the control group. Each of the voles was placed in the arena for 5 min per day without fighting with the other vole. Behavioral performance of two‐pair voles in the fighting experiment was classified into 4 types: combat, mild agonism, retreat, and amicable. By the end of this experiment, the whole fresh brains of all control group (*n* = 9) and the individuals that experienced intensive combat (voles wrestling, *n* = 9) were collected for neurobiological analysis.

### Real‐time PCR

Tissue specimens from bilateral brain regions, including the amygdala (AMYG), medial prefrontal cortex (MPOA), and paraventricular nucleus (PVN) were obtained by punching 200 μm brain sections from voles of different treatments. Total RNA was isolated according to the way of Trizol (Invitrogen, 1596‐026), and the collected RNA was dissolved in 40 μL DEPC buffer. First‐strand cDNA was synthesized using reverse transcriptase kit with Oligo (dt)18 primer (Fermentas, #K1622). The relative quantification of the OT, OTR, AVP, and AVPR gene was determined using SYBR Green PCR kit (Thermo, #K0223), and the expression of glyceraldehyde‐3‐phosphate dehydrogenase (GAPDH) was used to normalize the gene expression level (Applied Biosystems) according to the manufacturer's protocol. The data were analyzed by the 2^−∆∆Ct^ method. A standard curve was prepared by serial tenfold dilutions. The curve was linear over 7 logs with a correlation coefficient of 0.998. Primers and probes are designed for the *ot*, *otr*, *avp*, and *avpr* genes (Table S1, Supporting Information).

### Western blotting

Tissues from bilateral AMYG, MPOA, and PVN regions were obtained from 200 μm brain sections from voles of different treatment groups. Punch specimens were sonicated in 150−250 μL homogenization buffer per 20 mg tissue containing 1% sodium dodecyl sulfate (SDS), phosphatase inhibitor, and protease inhibitors (Beyotime Biotechnology, 5T533). The concentration of protein was determined using the BCA Protein Assay Kit (Thermo, PIPI2323), and 15 μL of total protein was loaded onto a 10% gradient Tris‐HCl polyacrylamide gel for electrophoresis fractionation (Bio‐Rad). Samples were transferred onto a nitrocellulose membrane (Millipore, HATF00010) and blocked in skim milk powder Blocking Buffer (BD, BYL40422) for 1 h except OT was blocked by SuperBlock T20 (TBS) Blocking Buffer (Thermo, 37536). After blocking, the same membrane was incubated at 4 °C overnight with either antibodies against OT (1:1500, Abcam, ab67457), OTR (1:3000, Abcam, ab181077), AVP (1:1000, SANTA, sc‐390702), AVPR (1:1000, Abcam, ab187753), or GAPDH (1:2000, CST, #5174) in skim milk powder blocking buffer or SuperBlock T20 (TBS) Blocking Buffer for OT. After thorough washing with Tris‐buffered saline and 0.1% Tween 20 (TBST) three times, blots were incubated for 1 h at a temperature of 37 °C with HRP secondary antibodies (1:1000; Beyotime Biotechnology, A0208/A0181/A0216) in skim milk powder or SuperBlock T20 (TBS) Blocking Buffer. Blots were developed by ECL TMB Substrate Solution (Millipore, WBKLS0100) and imaged with the Tanon Infrared Imaging system (Tanon−5200), then quantified by densitometry using ImageJ. The amount of protein blotted onto each lane was normalized to levels of GAPDH.

### Statistical analyses

A linear mixed model was also used to test the effect of housing density on the aggression behaviors; the housing density and days for behavioral recording were defined as fixed effect factors, and the cage was defined as a random effect factor. We used analysis of variance to test the significant effects of housing density, aggressive and crowding stresses on the expression levels of mRNA, and relevant protein for 4 genes (OT, OTR, AVP, and AVPR) in 3 brain areas (including AMYG, MPOA, and PVN). The normality assumption and the homogeneity of variance assumption were checked with the Shapiro–Wilk test and Levene's test, respectively. All statistical analysis was conducted in R (version 3.5.1).

## RESULTS

### Effects of housing density on behavior of Brandt's vole

**Figure 1:**
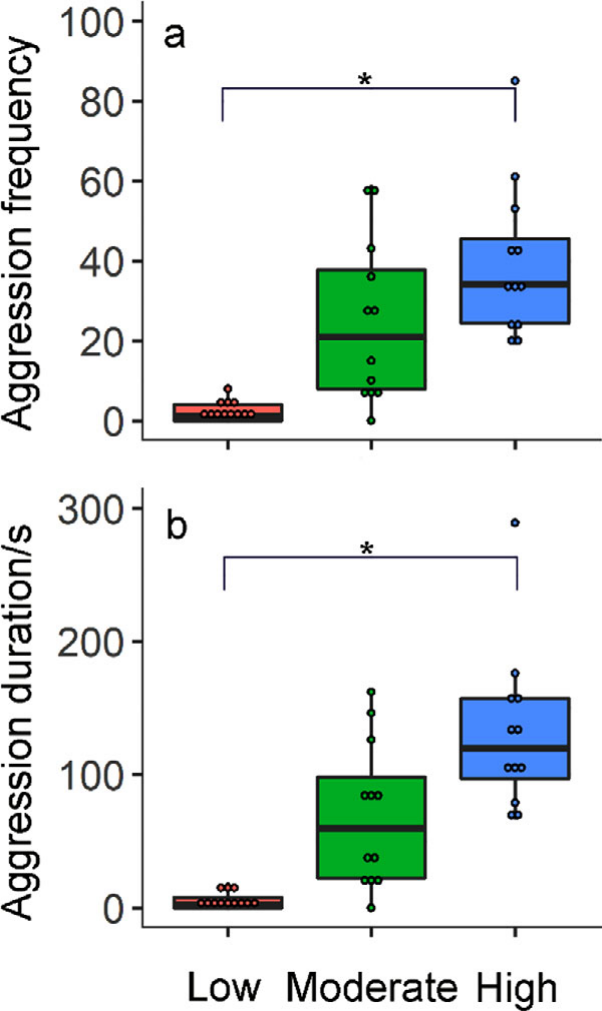
Effects of population density in housing density (a,b) on frequency of aggression or chasing behaviors between voles. Data are shown as mean ± SE. Asterisks indicate significant differences between density groups (^*^ *P* < 0.05, ^**^ *P* < 0.01, ^***^ *P* < 0.001). Low, Moderate, and High indicate the density group.

In the laboratory experiment, different housing densities significantly affected the aggression behaviors among voles (frequency: *F*_2,9_ = 9.3, *P* = 0.007; duration time: *F*_2,9_ = 12.5, *P* = 0.003). Voles housed at high density (8 voles/cage) showed more aggression behaviors than those housed at low density (2 voles/cage) (a 17 times increase in Frequency, Fig. 1a; a 23 times increase in duration time, Fig. 1b).

### Effect of housing density with physical contact on mRNA and protein expressions of OT/AVP systems

**Figure 2:**
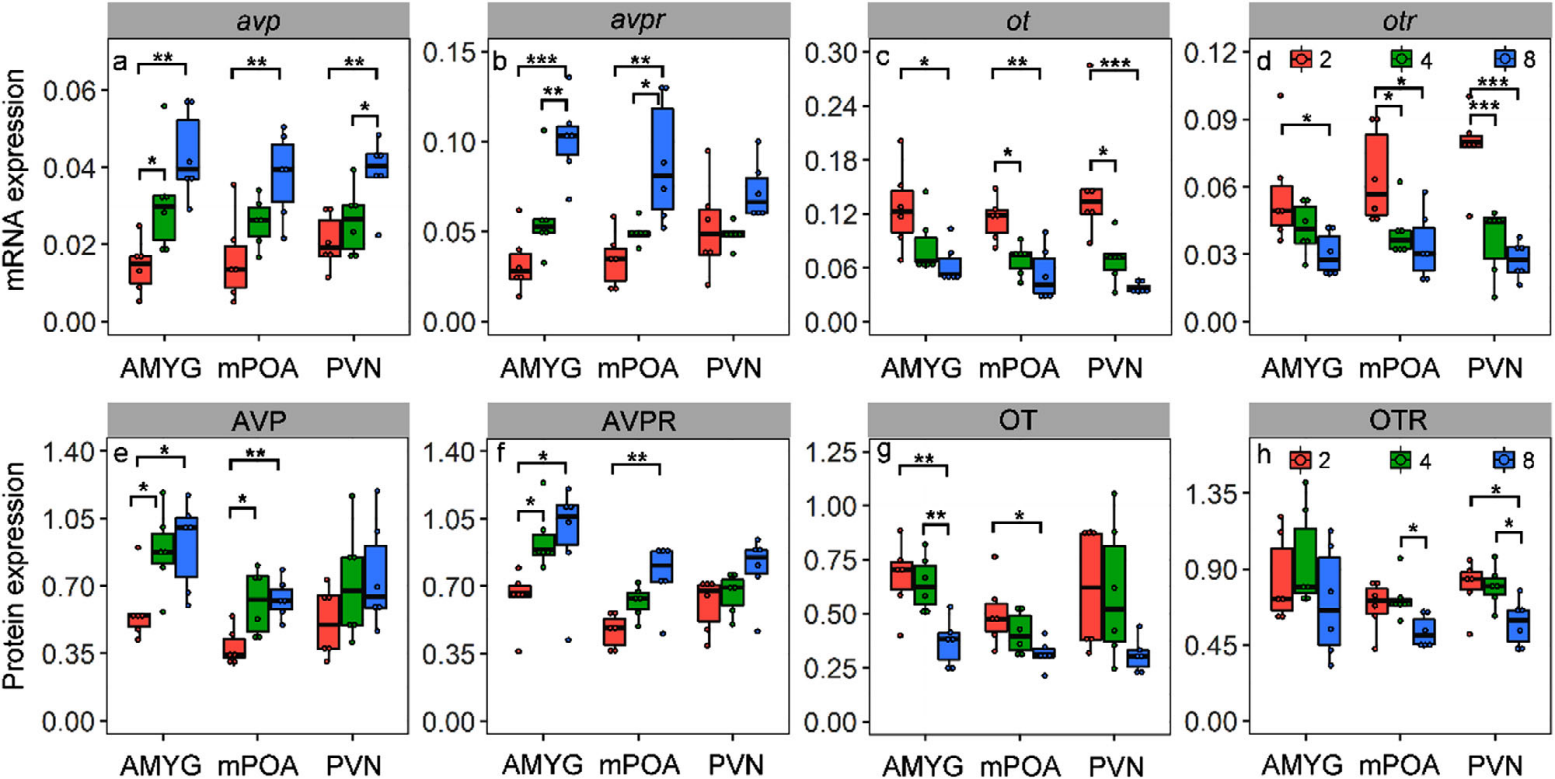
Effects of housing density (*n* = 2, 4, and 8 voles/cage) on the mRNA and protein expression of AVP, AVPR, OT, and OTR in different brain areas of AMYG, MPOA, and PVN. Data are shown as mean ± SE. Asterisks indicate significant differences between density groups (^*^ *P* < 0.05, ^**^ *P* < 0.01, ^***^ *P* < 0.001).

ANOVA results indicated that housing density showed significant and positive effects on the mRNA expression of AVP/AVPR (AVP: *F*_2,60_ = 19.6, *P* < 0.001; AVPR: *F*_2,60_ = 33.96, *P* < 0.001). In AMYG, MPOA, and PVN, voles in the high‐density group (8 voles/cage) all exhibited higher mRNA expression of AVP than those of low density group (2 voles/cage) (Fig. 2a; Table S2, Supporting Information). Similarly, mRNA expression of AVPR was also enhanced in voles housed at high‐density condition in all brain sites regions except for the PVN (Fig. 2b; Table S2, Supporting Information). Housing density showed significant and negative effects on mRNA expression of OT/OTR (OT: *F*_2,60_ = 21.2, *P* < 0.001; OTR: *F*_2,60_ = 33.96, *P* < 0.001). Low‐density groups (2 voles/cage) exhibited higher mRNA expression of OT/OTR than those of medium‐density group (4 voles/cage) and high‐density group (8 voles/cage) (Fig. 2c,d; Table S2, Supporting Information).

Housing density showed significant and positive effects on protein expressions of AVP/AVPR of voles (AVP: *F*_2,60_ = 7.12, *P* = 0.0017; AVPR: *F*_2,60_ = 13.12, *P* < 0.001). In both AMYG and MPOA regions, protein expressions of AVP/AVPR of medium‐density and high‐density groups were higher than that of low‐density group (Fig. 2e,f; Table S3, Supporting Information), whereas there was no effect of housing density on protein expressions of AVP/AVPR in PVN region. Housing density showed significant and negative effects on protein expression of OT/OTR (OT: *F*_2,60_ = 7.28, *P* = 0.0015; OTR: *F*_2,60_ = 9.42, *P* < 0.001). Protein expression of OT in AMYG and MPOA regions of low‐density group were all higher than that of high‐density group (AMYG: *t* = −3.88, *P* = 0.0015; MPOA: *t* = −2.93, *P* = 0.01). Protein expression of OT in PVN was not affected by housing density (Fig. 2g; Table S3, Supporting Information). Protein expression of OTR was significantly higher in low‐density or medium‐density groups than those in high‐density group for all brain regions except the AMYG (MPOA: *t* = −2.15, *P* = 0.048; PVN: *t* = −2.8, *P* = 0.014; Fig. 2h; Table S3, Supporting Information).

### Effect of housing density without physical contact on mRNA and protein expressions of OT/AVP systems

**Figure 3:**
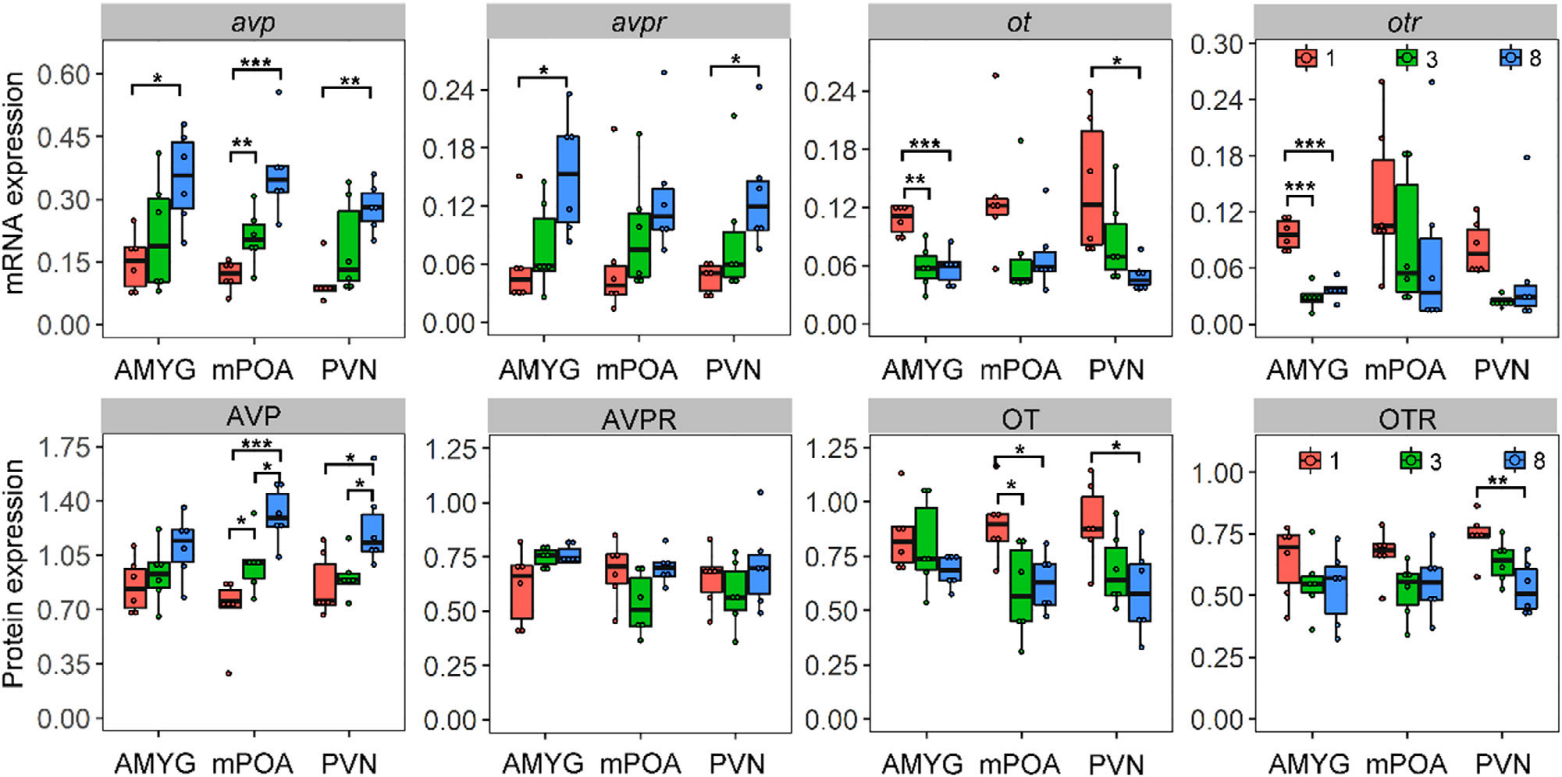
Effects of crowding stress on the mRNA and protein expression of AVP, AVPR, OT, and OTR in brain regions of AMYG, MPOA, and PVN of voles. Data are shown as mean ± SE. Asterisks indicate significant differences between groups (^*^ *P* < 0.05; ^**^ *P* < 0.01; ^***^ *P* < 0.001). 1, low‐crowding group with one neighbor; 3, medium‐crowding group with three neighbors; 8, high‐crowding group with eight neighbors.

In general, the higher density group without physical contact showed higher mRNA expression of AVP/AVPR than that of the lower density group in 3 brain regions, except for AVPR in MPOA (Fig. 3a,b; Table S4, Supporting Information). We found that high crowding density increases the protein expression of AVP in both MPOA and PVN (Fig. 3e; Table S5, Supporting Information). In contrast, the higher crowding density group showed lower mRNA or protein expression in OT/OTR than that of the lower‐density group in specific PVN or MPOA regions (Fig. 3c,d,g,h; Tables S4 and S5, Supporting Information).

### Effects of aggression stress on mRNA and protein expressions of OT/AVP systems

**Figure 4:**
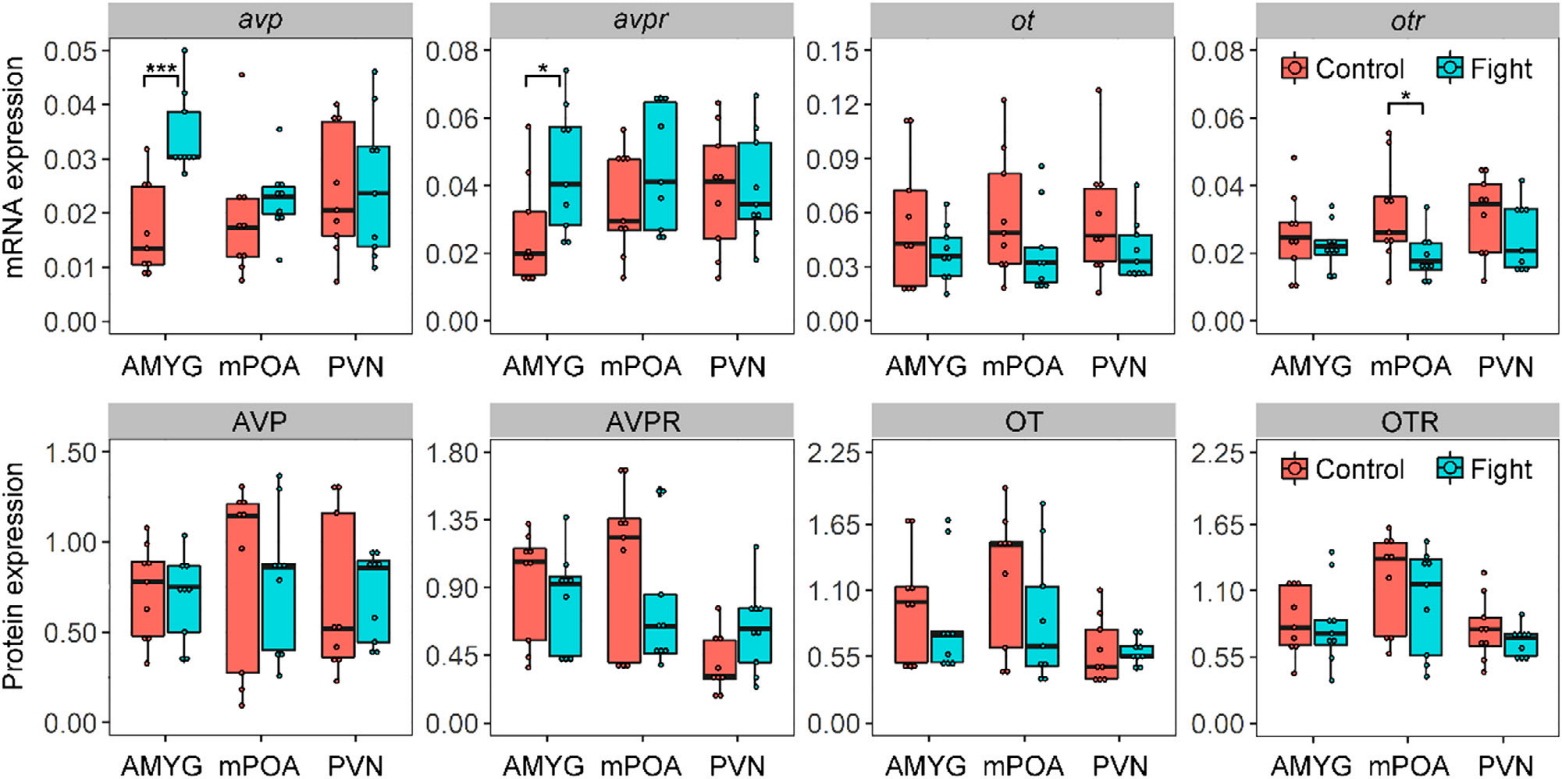
Effects of aggression stress on mRNA and protein expression of AVP, AVPR, OT, and OTR in different brain areas of AMYG, MPOA, and PVN of voles. Data are shown as mean ± SE. Asterisks indicate significant differences between groups (^*^ *P* < 0.05; ^**^ *P* < 0.01; ^***^ *P* < 0.001). Control, with no fighting; Fight, with fighting treatment between two voles.

Voles in the fighting group exhibited significant higher mRNA expression of AVP/AVPR in AMYG region than the control group; no such difference was found in MPOA and PVN regions (Fig. 4a,b). Three‐weeks fighting experience did not affect the protein expression of AVP/AVPR in AMYG, MPOA, and PVN (Fig. 4f). We found the fighting group exhibited a lower mRNA expression of OTR in MPOA region than that of control group, not the other brain regions (Fig. 4c,d). The protein expression of OT/OTR for Brandt's vole was not affected by the 3‐week fighting experience (Fig. 4g,h).

## DISCUSSION

Although neuropeptides AVP and OT have been implicated in stress responses and aggressive behaviors in a variety of mammalian species, the effects of density‐induced social stress on OT/AVP system has not been investigated. In this study, we firstly found that higher housing density with or without physical contact significantly increased mRNA and protein expression of AVP/AVPR but decreased mRNA and protein expression of OT/OTR in the specific brain regions of Brandt's voles. High housing density also increased the frequency and duration of aggressive behaviors. Furthermore, we demonstrated that aggressive behavior increased mRNA and protein expression of AVP/AVPR but decreased mRNA and protein expression of OT/OTR. Our results support our hypothesis that density‐induced social stress could alter OT/AVP systems through aggression with physical contact and crowding without physical contact. Because OT/AVP is known to regulate the behaviors of animals, we suggested that the observed density‐dependent effects on OT/AVP would play a significant role in regulating population dynamics of small rodents.

Many studies have shown that OT and its receptor (OTR) play a significant role in the regulation of social behaviors, including social affiliation, social memory, and recognition, as well as aggressive behavior (Tyzio *et al*. 2014; Hattori *et al*. 2015; Harony‐Nicolas *et al*. 2017). OT injection in the PVN can alleviate the stress response of the HPA axis by increasing secretion of local gamma‐aminobutyric acid (GABA), subsequently reducing the release of CRH and CORT as well as the defensive and aggressive behavior, thus promoting social affiliation and social bonding with other individuals (Smith & Wang 2012, 2014). The blockage of p‐Stat3‐Tyr^705^ signaling pathway in the nucleus accumbens will decrease the expression of OT and OTR, which not only increases aggressive behavior but also impairs social recognition (Yan *et al*. 2019), and OTR null mice display deficits in social behavior (Sala *et al*. 2011). Chronic intracerebroventricular (icv) infusion of OT in male rats reduced aggressive behaviors, whereas chronic infusion of the OTR antagonist increased their aggressive behavior (Calcagnoli *et al*. 2014). Low OT levels in cerebrospinal fluid may result in pathological aggression, and suicidal behavior in humans (Jokinen *et al*. 2012). In our previous study, we found administration of OT decreased aggression but separate administration of the OT antagonists and OTR antagonists significantly increased aggression of Brandt's voles (Yan *et al*. 2019). The AVP system has also been well recognized for its role in modulating aggression behaviors (Winslow *et al*. 1993). For example, injection of AVP within the ventrolateral hypothalamus enhanced offensive aggression in golden hamster (*Mesocricetus auratus*) (Delville *et al*. 1996). For monogamous prairie voles (*Microtus ochrogaster*), selective aggression associated with mate guarding was related to the increased activation of AVP‐expressing neurons in the AH (Gobrogge *et al*. 2007). It has also been shown that differences in AVP‐immunoreactive (AVP‐ir) staining in selected brain areas were associated with different attack latencies in the California mouse (*Peromyscus californicus*) (Bester‐Meredith & Marler 2001). Additionally, an increased level of AVPR receptor binding in the ventromedial hypothalamus was associated with the dominance status resulting from repeated agonistic encounters in male golden hamsters (Cooper *et al*. 2005).

In the present study, we found that, high population density was associated with increased frequency of aggression, enhanced levels of AVP/AVPR expression, and decreased levels of OT/OTR expression. These results support our hypothesis and our predictions (1) and (2), indicating that brain OT/AVP systems may play a significant role in mediating the density‐dependent aggressive behavior. Because of the strong associations of OT, AVP, and their receptor expression in the brain with changes in population density, we suggest that they can be used as a density‐dependency indicator in a population study of small rodents, which need to be verified in field studies.

Various environmental stressors could affect the brain OT/AVP systems. In rats, repeated restraint stress can facilitate the gene expression of AVP in the PVN. Forced swimming can also activate the release of AVP within the amygdala and hypothalamus in rats (Song et al. 1991). Chronic stress usually leads to the activation of the HPA axis that are characterized by the increases in plasma CORT and CRH gene expression in the hypothalamus, leading to anxiety/depression‐related behavior in both rats and humans (Mastorakos *et al*. 2006). Meynen has reported that AVP gene expression in the SON and PVN were significantly increased in major depression (Meynen *et al*. 2006). Similarly, previous research also found that mRNA levels of CRH and AVPR in the PVN were increased in the depressed patients (Wang *et al*. 2008). These studies concluded that OT/AVP systems play a major role in modulation of stress responses. Due to limited food resources and living space, high population density could enhance various environmental stressors (e.g. crowding or aggression), but it is not clear if the density‐dependent stress would alter the activity of OT/AVP systems, which regulates the aggressive behavior of animals. In this study, we found that high density increased AVP/AVPR expression but decreased the OT/OTR expression. Furthermore, we provided evidence that both aggression and crowding stress significantly caused the downregulation of the expression in OT/OTR but upregulation of AVP/AVPR in specific brain regions, which support our hypothesis and predictions (2) and (3). Our results suggest that the reciprocal enhancement between aggressive behavior and change of OT/AVP activities would be an important mechanism in mediating the aggression levels of rodents in high density conditions.

Our study indicated that high density as well as aggression could increase OT expression but decrease AVP expression in brains of voles. It is well established that decrease of OT or increase of AVP would greatly elevate the aggression behavior of animals. The reciprocal enhancement between aggressive behavior and changes of OT/AVP systems would further elevate the aggressive behaviors of animals in high‐density conditions. Our previous study indicated that change of AVP/OT could regulate the aggression of the Brandt's voles (Yan *et al*. 2019). There, we speculated that the density‐dependent association between OT/AVP and aggression would play a significant role in population regulation of small mammals. Future studies should be directed to test the observed density‐dependent relationship between OT/AVP system and aggression in field condition, and to examine the demographic consequences of these density‐dependent changes of OT/AVP in small rodents.

## ETHICAL STATEMENT

The study conforms to the legal requirements of China in which it was carried out, including those relating to conservation and welfare and to the journal's policy on these matters. The experimental protocols on animal behavioral experiments were consistent with regulations of the Institute of Zoology, Chinese Academy of Sciences. Ms. Shuli Huang and Mr. Jidong Zhao who conducted the animal behavioral experiments have been trained by the Beijing Agency for Experimental Animals, China, with authorized diploma.

## CONFLICT OF INTEREST

The authors declare no conflict of interest.

## AUTHOR CONTRIBUTIONS

Z.Z. designed this whole study; G.L. performed the density manipulation experiments in the lab; J.Z. performed the fighting experiment; S.H. performed the crowding experiment and all the neurobiological measurements of OT/AVP system; G.L., Z.W., and Z.Z. carried out the statistical analyses and plotting; S.H., G.L., and Z.Z. drafted the manuscript; Z.W., Y.P., M.S., X.W., C.K., and W.H. helped modify the manuscript. All authors gave final approval for publication.

## Supporting information


**Table S1** Sequences of the primers for qPCR experiments in this study
**Table S2** ANOVA results for the effects of housing density on mRNA expression in OT/AVP system for AMYG, mPOA and PVN
**Table S3** ANOVA results for the effects of housing density on protein expression in OT/AVP system for AMYG, mPOA and PVN
**Table S4** ANOVA results for the effects of crowding on mRNA expression in OT/AVP system for AMYG, mPOA and PVN
**Table S5** ANOVA results for the effects of crowding on protein expression in OT/AVP system for AMYG, mPOA and PVN
**Figure S1** Experimental design of laboratory housing density experiment. Voles could have physical contact with each other.
**Figure S2** Experimental design of laboratory crowding experiment. Voles were separated from each other by wire meshes. Voles could not have physical contact with each other, but they could communicate with each other via vision, hearing and odor.
Photo of Brandt's vole (*Lasiopodomys brandtii*)
