# Supplementary material for: Density‐induced social stress alters oxytocin and vasopressin activities in the brain of a small rodent species
Source: Integr Zool. 2020 Aug 16;16(2):149–59. doi: 10.1111/1749-4877.12467 (PMC7891312; doi:10.1111/1749-4877.12467)
Supplement: Supplementary file 1 — Table S1 Sequences of the primers for qPCR experiments in this study Table S2 ANOVA results for the effects of housing density on mRNA expression in OT/AVP system for AMYG, mPOA and PVN Table S3 ANOVA results for the effects of housing density on protein expression in OT/AVP system for AMYG, mPOA and PVN Table S4 ANOVA results for the effects of crowding on mRNA expression in OT/AVP system for AMYG, mPOA and PVN Table S5 ANOVA results for the effects of crowding on protein expression in OT/AVP system for AMYG, mPOA and PVN Figure S1 Experimental design of laboratory housing density experiment. Voles could have physical contact with each other. Figure S2 Experimental design of laboratory crowding experiment. Voles were separated from each other by wire meshes. Voles could not have physical contact with each other, but they could communicate with each other via vision, hearing and odor. Photo of Brandt's vole (Lasiopodomys brandtii) [file INZ2-16-149-s001.doc]

**SUPPLEMENTARY MATERIALS**

**Table S1** Sequences of the primers for qPCR experiments in this study

| Gene | Primers | Sequence(5'- 3') |
| --- | --- | --- |
| Ot | Forward | TGCCAGGAGGAGAACTACC |
|  | Reverse | TCCGAGAAGGCAGACTCAG |
| Otr | Forward | CTCCCACCTATTTCTACTACC |
|  | Reverse | TCATTTCCCACTCCTTGTC |
| Avp | Forward | ACGCTCTCCGCTTGTTTC |
|  | Reverse | ACGCTCTCCGCTTGTTTC |
| V1aR | Forward | ATGGCACCAAAGCCCAAG |
|  | Reverse | ACAAGGCGTGACCAGAAG |
| Gapdh | Forward | ATCACTGCCACCCAGAAG |
|  | Reverse | TCCACGACGGACACATTG |

**Table S2** ANOVA results for the effects of housing density on mRNA expression in OT/AVP system for AMYG, mPOA and PVN. M: medium density group; L: low density group; H: high density group.

| Gene | Brain area | **Comparisons of mRNA expression** | *t* value | *p* value | Overall F value | Overall *P* value |
| --- | --- | --- | --- | --- | --- | --- |
| ot | AMYG | M vs L | −2.1 | 0.056 | 4.98 | 0.022 |
|  |  | H vs L | −3.1 | 0.0073 |  |  |
|  | mPOA | M vs L | −3.2 | 0.0054 | 10.5 | 0.0014 |
|  |  | H vs L | −4.4 | 0.0005 |  |  |
|  | PVN | M vs L | −3.3 | 0.0046 | 11.2 | 0.001 |
|  |  | H vs L | −4.6 | 0.00036 |  |  |
| otr | AMYG | M vs L | −1.6 | 0.12 | 4.1 | 0.038 |
|  |  | H vs L | −2.85 | 0.012 |  |  |
|  | mPOA | M vs L | −2.55 | 0.022 | 5.77 | 0.014 |
|  |  | H vs L | −3.2 | 0.0057 |  |  |
|  | PVN | M vs L | −5.1 | 0.0001 | 21.54 | 0.00004 |
|  |  | H vs L | −6.1 | 0.00002 |  |  |
| avp | AMYG | M vs L | 2.6 | 0.02 | 10.35 | 0.0015 |
|  |  | H vs L | 4.5 | 0.0004 |  |  |
|  | mPOA | M vs L | 1.72 | 0.1 | 7.51 | 0.005 |
|  |  | H vs L | 3.87 | 0.0015 |  |  |
|  | PVN | M vs L | 1.11 | 0.28 | 7.42 | 0.006 |
|  |  | H vs L | 3.75 | 0.002 |  |  |
| avpr | AMYG | M vs L | 2.1 | 0.053 | 15.6 | 0.0002 |
|  |  | H vs L | 5.5 | 0.00006 |  |  |
|  | mPOA | M vs L | 1.16 | 0.026 | 9.84 | 0.0018 |
|  |  | H vs L | 4.29 | 0.0006 |  |  |
|  | PVN | M vs L | −0.37 | 0.7 | 3.05 | 0.08 |
|  |  | H vs L | 1.93 | 0.07 |  |  |

**Table S3** ANOVA results for the effects of housing density on protein expression in OT/AVP system for AMYG, mPOA and PVN. M: medium density group; L: low density group; H: high density group.

| Gene | Brain area | **Comparisons of** **protein expression** | t value | p value | Overall F value | Overall P value |
| --- | --- | --- | --- | --- | --- | --- |
| ot | AMYG | M vs L | −0.41 | 0.69 | 9.1 | 0.0026 |
|  |  | H vs L | −3.88 | 0.0015 |  |  |
|  | mPOA | M vs L | −1.4 | 0.17 | 4.3 | 0.033 |
|  |  | H vs L | −2.93 | 0.01 |  |  |
|  | PVN | M vs L | −0.16 | 0.87 | 2.89 | 0.08 |
|  |  | H vs L | −2 | 0.057 |  |  |
| otr | AMYG | M vs L | 0.67 | 0.51 | 1.04 | 0.38 |
|  |  | H vs L | −0.77 | 0.45 |  |  |
|  | mPOA | M vs L | 0.64 | 0.53 | 4.28 | 0.034 |
|  |  | H vs L | −2.15 | 0.048 |  |  |
|  | PVN | M vs L | −0.04 | 0.97 | 5.08 | 0.02 |
|  |  | H vs L | −2.78 | 0.014 |  |  |
| avp | AMYG | M vs L | 2.64 | 0.018 | 5.19 | 0.02 |
|  |  | H vs L | 2.92 | 0.01 |  |  |
|  | mPOA | M vs L | 3.2 | 0.006 | 7.35 | 0.006 |
|  |  | H vs L | 3.4 | 0.0037 |  |  |
|  | PVN | M vs L | 1.35 | 0.19 | 1.52 | 0.25 |
|  |  | H vs L | 1.63 | 0.12 |  |  |
| avpr | AMYG | M vs L | 2.55 | 0.022 | 4.56 | 0.028 |
|  |  | H vs L | 2.68 | 0.017 |  |  |
|  | mPOA | M vs L | 2.2 | 0.046 | 8.8 | 0.003 |
|  |  | H vs L | 4.2 | 0.00077 |  |  |
|  | PVN | M vs L | 0.66 | 0.52 | 2.61 | 0.11 |
|  |  | H vs L | 1.9 | 0.07 |  |  |

**Table S4** ANOVA results for the effects of crowding on mRNA expression in OT/AVP system for AMYG, mPOA and PVN. M: medium crowing group; L: low crowding group; H: high crowding group.

| Gene | Brain area | **Comparisons of mRNA expression** | t value | p value | Overall F value | Overall P value |
| --- | --- | --- | --- | --- | --- | --- |
| ot | AMYG | M vs L | −4.5 | 0.0004 | 13.85 | 0.0004 |
|  |  | H vs L | −4.6 | 0.00035 |  |  |
|  | mPOA | M vs L | −1.9 | 0.076 | 2.51 | 0.11 |
|  |  | H vs L | −1.97 | 0.067 |  |  |
|  | PVN | M vs L | −1.97 | 0.067 | 5.3 | 0.018 |
|  |  | H vs L | −3.2 | 0.0056 |  |  |
| otr | AMYG | M vs L | −8.6 | 0.00000035 | 43.8 | 0.0000005 |
|  |  | H vs L | −7.5 | 0.000002 |  |  |
|  | mPOA | M vs L | −0.94 | 0.36 | 0.79 | 0.47 |
|  |  | H vs L | −1.2 | 0.25 |  |  |
|  | PVN | M vs L | −2.4 | 0.03 | 2.89 | 0.08 |
|  |  | H vs L | −1.28 | 0.22 |  |  |
| avp | AMYG | M vs L | 1 | 0.33 | 5.33 | 0.018 |
|  |  | H vs L | 3.19 | 0.006 |  |  |
|  | mPOA | M vs L | 2.1 | 0.055 | 16.4 | 0.00017 |
|  |  | H vs L | 5.66 | 0.00004 |  |  |
|  | PVN | M vs L | 1.81 | 0.09 | 7.89 | 0.0045 |
|  |  | H vs L | 3.97 | 0.001 |  |  |
| avpr | AMYG | M vs L | 0.62 | 0.55 | 5.51 | 0.016 |
|  |  | H vs L | 3.13 | 0.0068 |  |  |
|  | mPOA | M vs L | 0.74 | 0.47 | 1.67 | 0.22 |
|  |  | H vs L | 1.82 | 0.089 |  |  |
|  | PVN | M vs L | 1.35 | 0.196 | 4.17 | 0.036 |
|  |  | H vs L | 2.88 | 0.011 |  |  |

**Table S5** ANOVA results for the effects of crowding on protein expression in OT/AVP system for AMYG, mPOA and PVN. M: medium crowding group; L: low crowding group; H: high crowding group.

| Gene | Brain area | **Comparisons of** **protein expression** | t value | p value | Overall F value | Overall P value |
| --- | --- | --- | --- | --- | --- | --- |
| ot | AMYG | M vs L | −0.47 | 0.64 | 1.6 | 0.23 |
|  |  | H vs L | −1.7 | 0.1 |  |  |
|  | mPOA | M vs L | −3.1 | 0.007 | 5.67 | 0.015 |
|  |  | H vs L | −2.69 | 0.017 |  |  |
|  | PVN | M vs L | −2 | 0.06 | 4.5 | 0.029 |
|  |  | H vs L | −2.9 | 0.01 |  |  |
| otr | AMYG | M vs L | −1.08 | 0.29 | 0.94 | 0.4 |
|  |  | H vs L | −1.27 | 0.22 |  |  |
|  | mPOA | M vs L | −2.12 | 0.047 | 2.63 | 0.1 |
|  |  | H vs L | −1.73 | 0.1 |  |  |
|  | PVN | M vs L | −1.85 | 0.08 | 7.1 | 0.0068 |
|  |  | H vs L | −3.77 | 0.0018 |  |  |
| avp | AMYG | M vs L | 0.68 | 0.5 | 2.72 | 0.098 |
|  |  | H vs L | 2.27 | 0.038 |  |  |
|  | mPOA | M vs L | 2.63 | 0.019 | 14.69 | 0.0003 |
|  |  | H vs L | 5.4 | 0.00007 |  |  |
|  | PVN | M vs L | 2.522 | 0.023 | 5.77 | 0.014 |
|  |  | H vs L | 3.17 | 0.006 |  |  |
| avpr | AMYG | M vs L | 2.24 | 0.04 | 3.67 | 0.05 |
|  |  | H vs L | 2.44 | 0.0278 |  |  |
|  | mPOA | M vs L | −2.12 | 0.051 | 3.42 | 0.059 |
|  |  | H vs L | 0.26 | 0.79 |  |  |
|  | PVN | M vs L | −0.79 | 0.44 | 0.99 | 0.39 |
|  |  | H vs L | 0.61 | 0.55 |  |  |


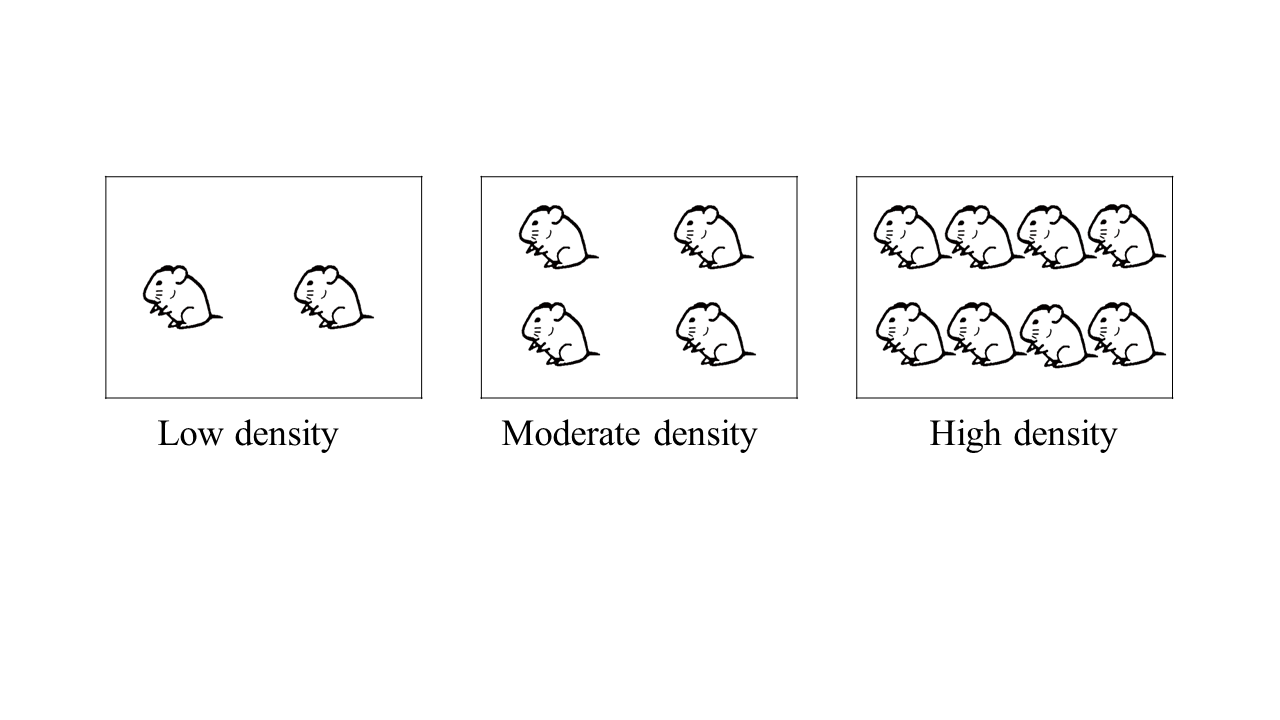


**Figure S1** Experimental design of laboratory housing density experiment. Voles could have physical contact with each other.


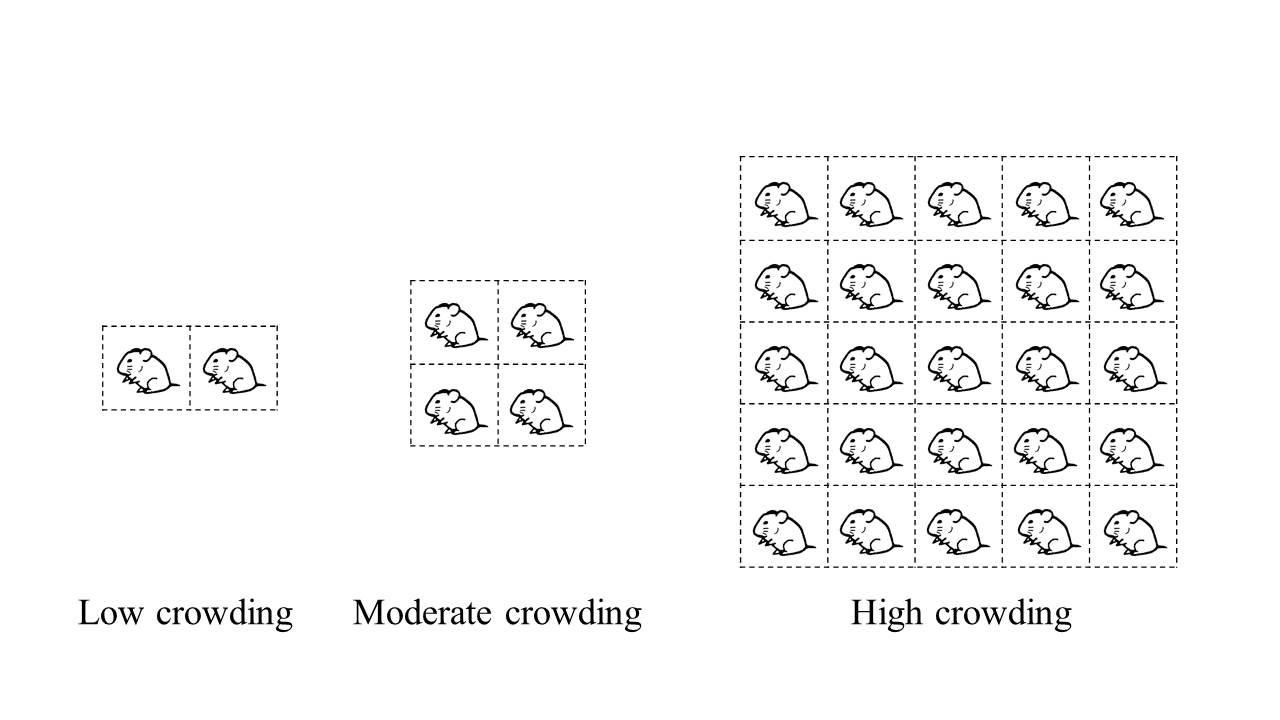


**Figure S2** Experimental design of laboratory crowding experiment. Voles were separated from each other by wire meshes. Voles could not have physical contact with each other, but they could communicate with each other via vision, hearing and odor.


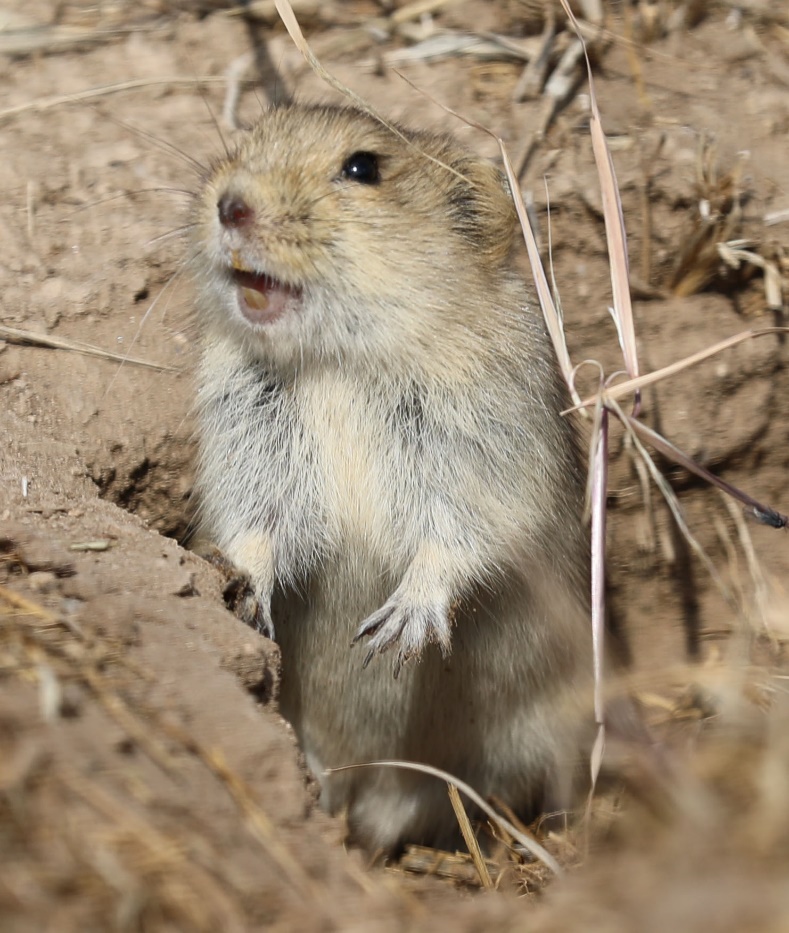


Photo of Brandt’s vole (*Lasiopodomys brandtii*) taken by Guoliang Li
